# Alternating hemiplegia of childhood and rapid-onset dystonia-parkinsonism are both ATP1A3-related disorders

**DOI:** 10.1186/2194-7791-1-S1-A15

**Published:** 2014-09-11

**Authors:** Hendrik Rosewich, Holger Thiele, Andreas Ohlenbusch, Ulrike Maschke, Peter Frommolt, Peter Nürnberg, Knut Brockmann, Jutta Gärtner

**Affiliations:** Department of Paediatrics and Paediatric Neurology, University Medical Center Göttingen, Georg August University Göttingen, Göttingen, Germany; Cologne Center for Genomics, University of Cologne, Cologne, Germany; Catholic Hospital St Johann Nepomuk, Erfurt, Germany; Department of Pediatrics and Adolescent Medicine, University Medical Center Göttingen, Georg August University Göttingen, Göttingen, Germany

Alternating hemiplegia of childhood (AHC) was first described as a distinctive disease in 1971 [1]. The disease is characterised by early-onset episodes of hemiplegia, dystonia, numerous paroxysmal symptoms, and developmental impairment [2]. Almost all cases of AHC are sporadic.

To identify *de-novo* mutations associated with this disease 40 clinically well-characterized patients were recruited from September 2004 till April 2013. Whole-exome sequencing was performed in three proband-parent trios. Informative genes were evaluated in the 37 remaining patients and *ATP1A3* emerged as the gene associated with AHC [3]. Interestingly, this gene was already known to be associated with another movement disorder with later onset namely rapid-onset dystonia-parkinsonism (RDP) [4]. We then thoroughly analysed clinical and molecular findings of AHC and RDP to evaluate the phenotypic and genotypic spectrum. In addition, we started to analyse the functional consequences of the encoded Na^+^,K^+^ alpha 3 subunit for different *ATP1A3* mutations associated with either AHC or RDP applying cell survival assays and two-electrode voltage clamp techniques.

39 of 40 patients with a characteristic AHC/RDP phenotype displayed a *de-novo* mutation in *ATP1A3*. Our study first showed that AHC and RDP are not two distinct diseases but rather constitute a clinical continuum of one disorder with AHC at the severe end of the spectrum and RDP as a milder variant. Clinically overlapping features are abrupt onset of triggered dystonic episodes, a rostrocaudal gradient of involvement as well as brainstem dysfunction; clearly differentiating characteristics are fixed dystonia in RDP and episodic hemiplegia in AHC. Further, mutations affecting functional and transmembrane protein domains tend to be associated with an AHC phenotype and the majority of *ATP1A3* mutations are located in only four exons [5]. To further elucidate the pathomechanisms of ATP1A3 related disorders *in vitro* studies on selected mutations are ongoing.
